# Role of Lectin in the Response of *Aedes aegypti* Against *Bt* Toxin

**DOI:** 10.3389/fimmu.2022.898198

**Published:** 2022-05-13

**Authors:** Intikhab Alam, Khadija Batool, Aisha Lawan Idris, Weilong Tan, Xiong Guan, Lingling Zhang

**Affiliations:** ^1^State Key Laboratory of Ecological Pest Control for Fujian and Taiwan Crops, College of Life Sciences, Fujian Agriculture and Forestry University, Fuzhou, China; ^2^Key Lab of Biopesticides and Chemical Biology, MOE, Fujian Agriculture and Forestry University, Fuzhou, China; ^3^College of Life Sciences, South China Agricultural University, Guangzhou, China; ^4^Nanjing Bioengineering (Gene) Technology Center for Medicines, Nanjing, China

**Keywords:** *Bacillus thuringiensis*, *Aedes aegypti*, lectin, toxicity, mechanism

## Abstract

*Aedes aegypti* is one of the world’s most dangerous mosquitoes, and a vector of diseases such as dengue fever, chikungunya virus, yellow fever, and Zika virus disease. Currently, a major global challenge is the scarcity of antiviral medicine and vaccine for arboviruses. *Bacillus thuringiensis* var israelensis (*Bti*) toxins are used as biological mosquito control agents. Endotoxins, including Cry4Aa, Cry4Ba, Cry10Aa, Cry11Aa, and Cyt1Aa, are toxic to mosquitoes. Insect eradication by Cry toxin relies primarily on the interaction of cry toxins with key toxin receptors, such as aminopeptidase (APN), alkaline phosphatase (ALP), cadherin (CAD), and ATP-binding cassette transporters. The carbohydrate recognition domains (CRDs) of lectins and domains II and III of Cry toxins share similar structural folds, suggesting that midgut proteins, such as C-type lectins (CTLs), may interfere with interactions among Cry toxins and receptors by binding to both and alter Cry toxicity. In the present review, we summarize the functional role of C-type lectins in *Ae. aegypti* mosquitoes and the mechanism underlying the alteration of Cry toxin activity by CTLs. Furthermore, we outline future research directions on elucidating the *Bti* resistance mechanism. This study provides a basis for understanding *Bti* resistance, which can be used to develop novel insecticides.

## Introduction

The mosquito *Aedes aegypti* is one of the most important species responsible for transmitting viruses that cause life-threatening and epidemic human diseases worldwide, such as dengue virus (DENV), yellow fever virus (YFV), chikungunya virus (CHIKV) and, Zika virus (ZIKV), which drastically affect human populations (1). Dengue fever is a rapidly spreading arbovirus that has become a global health concern (2). The rapid expansion of CHIKV and ZIKV demands the identification of circulating lineages to design effective surveillance programs. The main vectors for the spread of these viruses in urban areas are *Ae. aegypti* (L.) and *Ae. albopictus* (Skuse), although other mosquito species have also been reported (3–5). To date, no efficient antiviral drugs or vaccines have been developed to control these viral diseases, with the exception of yellow fever. As a necessary consequence, efforts to control mosquito populations remain a critical strategy for reducing infection rates.

Chemical insecticides with active components, such as organophosphates, pyrethroids, organochlorines, and carbamates, have been used to control these disease vectors (6, 7). However, these chemicals are damaging to both the natural environment and human health. They cause depletion of natural enemies in the ecosystem and the development of insect resistance when used continuously (8–10). In recent years, chemical insecticides have been successfully replaced by eco-friendly biological control agents with high specificity, minimal influence on non-target organisms, and reduced insect resistance (11–13). Entomopathogenic bacteria, such as *Bacillus thuringiensis* (*Bt*), which produce different toxin spores, represent a promising substitute for mosquito control. These bacterial spores have a high potential to control insect pests (14–16) (17). *Bt* produces a number of crystal proteins that have insecticidal activity against over 3000 insect species, including Coleoptera, Lepidoptera, and Dipterans (18–20). These toxin proteins, including Cry4Aa, Cry4Ba, Cry10Aa, Cry11Aa, and Cyt1Aa, are toxic to mosquitoes (21–23). Cry toxin’s effectiveness against insect pests is dependent on their interactions with other receptors such as alkaline phosphatase (ALP), aminopeptidase-N (APN), cadherin (CAD), and ATP-binding cassette (ABC) transporters (24–31). For that reason, it is crucial to comprehend the interactions between Cry toxins and other midgut proteins. In addition to Cry toxins, Cyt toxins are important for inducing toxicity in some insect orders (23, 32, 33). For example, the *Bt* strain LLP29 produces the Cyt1Aa6 toxin, which is toxic to *Ae. albopictus* and *Culex quinquefasciatus* (34).

Lectins are a diverse group of ubiquitous carbohydrate-binding proteins found in all organisms that play an important role in self/non-self-immune recognition in insects (35–40). Lectins have a wide range of functional responses in symbiosis, host colonization by microbial pathogens, and host immune responses (41, 42). Genome-wide analyses have shown that C-type lectin (CTL) proteins are more abundant and distinct in invertebrates (43–47). Moreover, lectin proteins bind carbohydrates in the existence of Ca^2+^ ions *via* their C-type lectin-like domains (CTLD), containing the highly conserved motifs QPD (Gln-Pro-Asp) and, EPN (Glu-Pro-Asn) which are specific to mannose- and galactose-type carbohydrates (43). The Cry toxin domains II and III and carbohydrate-recognition domains (CRD) of lectins have similar structures (48–50), and because of these structural similarities, it is very important to further functionally investigate and comprehend the role and functional mechanism of lectins in Cry toxicity. Protein-protein interactions among lectin, Cry toxin, and related toxin receptors have been investigated to explore the function of lectin in *Bt* serovar *israelensis* (*Bti*) tolerance (51–54). Lectin binding research also showed the existence of numerous APN isoforms with O-linked carbohydrate structures known to bind with Cry1Ac toxin in Douglas fir tussock moth larvae (55). The lectin-like domain III of Cry toxins also known to involved in the interaction with the peritrophic membrane (PM) by attaching to PM chitin and GalNAc related numerous PM proteins (56–58), which may also contribute to the failure of some toxins to pass through the PM (59, 60). However, understanding the role of lectins in Cry toxicity is important, as it will not only broaden our understanding of the *Bt* mechanism but also aid in the implementation of new biocontrol strategies.

## *Ae. aegypti* Invasion

*Ae. aegypti* is an important arthropod vector and model organism in invasion biology. Competition for the same available resources in the ecosystem disrupts and destabilizes the native population (61). nvasion results in the introduction of new diseases or the active spread of local diseases. Mosquitoes are important invaders due to a close relationship with human pathogens (62, 63). Human habitats are the most likely places for mosquitoes to live in and most mosquitoes change territories accordingly (64). *Ae. aegypti* survive worldwide in tropical and subtropical areas; however, populations vary in their capability (vector capacity) to transmit disease (65–70). Africa is considered the ancestral location of *Ae. aegypti*, which spread to other parts of the world probably by traveling on ships along trading routes (67, 69). Outside Africa, *Ae. aegypti* has a robust genetic inclination to enter homes and feed on humans’ blood, as well as the ability to survive and lay eggs in man-made water reservoirs in the human environment (66, 70). However, there is extensive variation in the appearance, ecology, and behavior among sub-Saharan African mosquito populations (6, 10, 71–73). Some populations are less contact with humans, live in forests, feed on other animals, and oviposit in tree holes (66, 67, 69, 70).

### Origin of *Ae. aegypti*

There are two subspecies of *Ae. aegypti* (69), namely, *Ae. aegypti formosus* (Aaf) and *Ae. aegypti aegypti (Aaa)*. Almost all populations of the African subspecies *Ae. aegypti aegypti* are strongly anthropophilic and light in color. However, in Africa, subspecies belonging to the *Ae. aegypti formosus* live in forests and are darker in color. Previously, the two subspecies were separated by coloration, with *Aedes aegypti aegypti* having pale scales on the first abdominal tergite (69). However, the populations of West Africa contain pale scales, on the other hand, appeared to be closer genetically to *Aedes aegypti formosus* populations than to *Aedes aegypti aegypti* populations from other parts of the tropics (10, 72, 73). Both species coexist in West Africa (Senegal) and East Africa (Kenya). Although they do not coexist in rural areas, they mate freely in urban environments. The combination of different factors, such as low migration, founder effects, and irregular habitats, makes populations more genetically structured (74). In earlier 16^th^ to 18^th^ centuries, trans-Atlantic shipping introduced *Ae. aegypti* to the recent world and in the late 19^th^ century *Ae. aegypti* reached Asia (75–77). The mosquito exomes from five different populations of the globe were sequenced and compared them with those of the African populations of *Ae. aegypti* in West Africa (Senegal) and other regions (Mexico and Sri Lanka) (78).

### *Ae. aegypti* Biology

**Figure 1:**
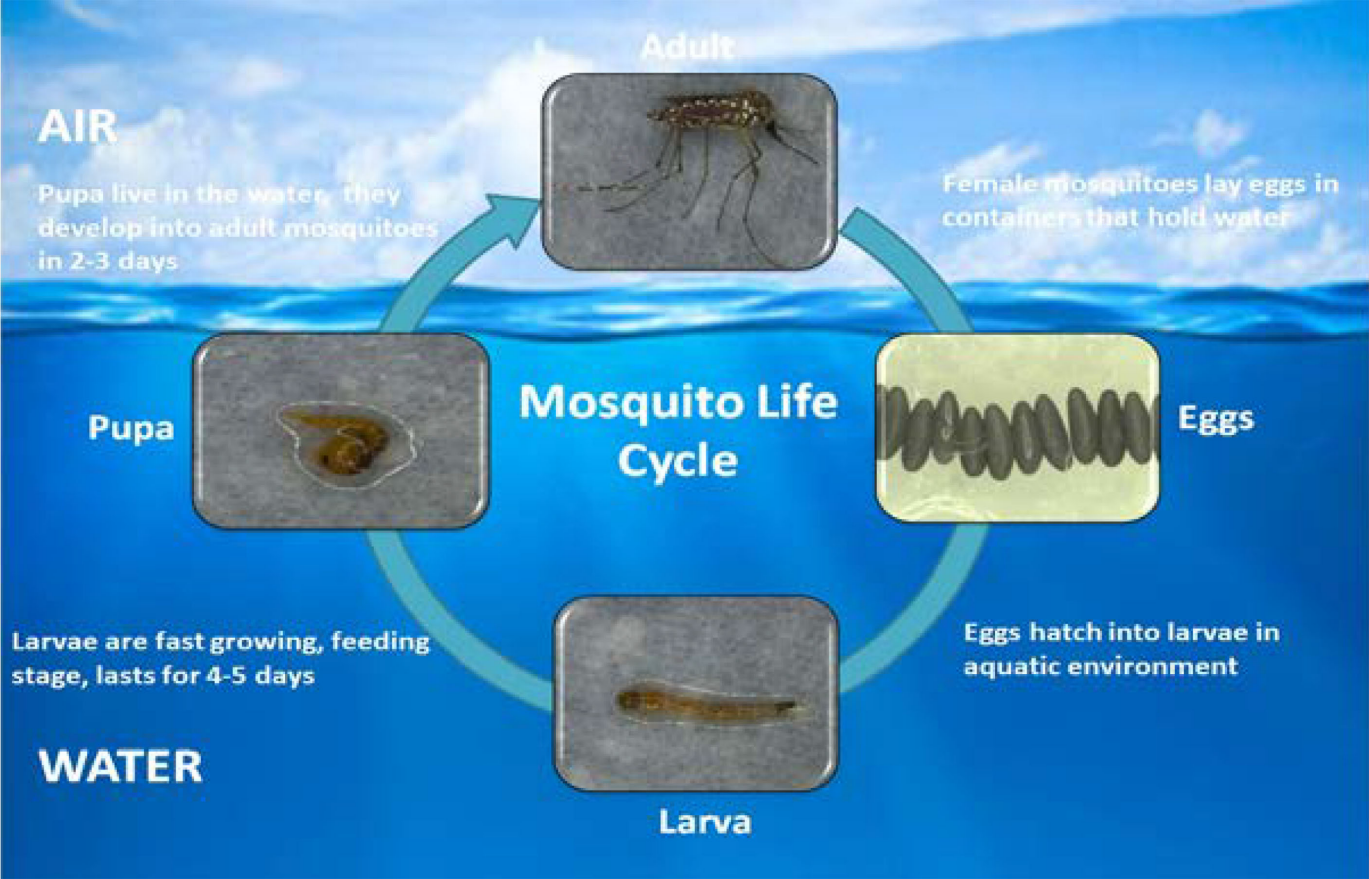
Life cycle of mosquito *Aedes aegypti*.

Generally, plant nectar acts as a basic source of food for mosquitoes, but female mosquitoes require blood prior to laying eggs. Warm-blooded vertebrate host blood is a preferred nutrient source for adult female mosquitoes (79). Humans are the most stable hosts for sucking blood. Nutrients in the larval stages are stored and consumed during egg production (80). During its lifespan, an adult female can lay five batches of eggs, with a single batch containing up to 100-200 eggs. Eggs can resist drought conditions for a few months (81, 82). Most parts of the mosquito life cycle are in the aquatic phase, including the four larval stages and pupal stage. Larvae are fast growing, feeding completely on the water surface. The larval stages last for at least four days. At the end of the fourth instar, the larvae go through a non-feeding stage called the pupal phase, which lasts approximately two days. The lifespan of an adult mosquito changes according to environmental circumstances but generally ranges from two to four weeks (**Figure 1**) (81, 83, 84).

## Global Burden of Mosquito-Borne Diseases

Vector-borne diseases affect two-thirds of the world’s population and cause the death of millions of people annually (66, 85, 86). *Ae. aegypti* is the main arboviruses vector (87–89). It is mainly linked with the spread of a many viral diseases in humans, including dengue fever, yellow fever, chikungunya and Zika virus disease. However, the world is less affected by yellow fever as a potent vaccine has been developed to control it, although it still exists (90, 91). Dengue viruses (DENVs) are the causal agents of dengue fever, a viral infectious mosquito-borne disease that spreads across the world’s tropics and subtropics (92). There are four DENV serotypes, namely, DENV-1, DENV-2, DENV-3, and DENV-4 (93, 94). Each year, approximately 390 million people worldwide become infected with the dengue virus (95). In 2014, the highest spread of dengue fever occurred in Taiwan with 15,732 reported cases, of which 136 were dengue hemorrhagic fever (96). From 1990 to 2019, the burden of dengue increased as most parts of the world experienced three decades of urbanization, global warming, and an increased population. Southeast Asia and South Asia remain areas of concern, especially as the burden of dengue fever in the Americas is rapidly increasing (97).

In 2007, the Zika virus (ZIKV) was detected in 55 countries in America, Oceania, Asia, and Africa. However, the first epidemic cases were recorded in Brazil in 2015 and approximately 1.5 million people were infected (98). Zika virus disease, which results in microcephaly in newborns, affects brain growth, and leads to the formation of cranial calcifications, is becoming increasingly prevalent in Brazil (99). An outbreak of Zika virus disease in South America, Central America, and the Caribbean was linked to prenatal brain dysfunction (100). The chikungunya virus (CHIKV) belongs to the Alphavirus genus, which is transmitted by both *Ae. aegypti* and *Ae. albopictus*, causing chikungunya fever with serious joint pain in infected patients for several years (101). In 1952-1953 the first CHIKV epidemic was reported in Tanzania (East Africa) (101) and considered as a leading reason of concern, causing epidemics in several Indian Ocean islands, Asia, as well as in America and Southern Europe. In 2005-2006, a CHIKV epidemic outbreak occurred in the Indian Ocean and 1.5 million people were infected. In 2010, an epidemic outbreak was reported in India, affecting more than one million people (102). However in 2013, CHIKV spread in the Western world and further spread in the Americas (46 countries) and 1.7 million suspected cases were reported (103). Existing data show that between 2010 and 2019, CHIKV and ZIKV caused average annual losses of more than 106,000 and 44,000 disability-adjusted life years (DALYs), respectively. The burden of these two viruses in the Americas far exceeds that of any other region of the World Health Organization (WHO) (104).

## Biocontrol of Mosquitoes Using *B. thuringiensis*

The discovery of bacteria such as *Bti* are extremely toxic to Dipteran larvae, has opened the way to their usage as a possible bio-larvicide in mosquito eradication campaigns across the world (22, 105, 106). *Bti* toxin was initially found to be an excellent biological control agent for mosquito larvae and black flies (107). It can produce different toxins, such as Cry4Aa, Cry4Ba, Cry11Aa, Cyt1Aa, and Cyt2Ba crystal proteins (108, 109). Cry proteins are known to be very toxic against different insect orders, such as Coleopteran, Diptera, Lepidoptera, and Hymenoptera. In contrast, Cyt toxins are usually found in *Bt* strains that are active against Dipterans, with a few outliers of Cyt proteins that, are active against Coleopteran larvae have been documented (32, 110). However, Cry11Aa exhibited a high toxicity against *Ae. aegypti* (111). At present, *Bti* is largely used for mosquito control; therefore, improving the effectiveness of *Bti* products is a key issue that needs to be solved in the current development of *Bti* products. Biocontrol product limitations can be improved by enhancing the genetic and physiological mechanisms of biocontrol using a mixture of organisms as biocontrol agents (112, 113).

According to all the known Cry structures, activated Cry toxins have three individual functional domains consisting of α-helical bundles in domain-I, β-prism folds in domain-II, and a sandwich of αβ-sheets in domain-III. Domains I and II function in receptor recognition and membrane pore formation, respectively (114). Cry toxins interact with midgut receptors found in lipid rafts and this phase is necessary for oligomerization and toxin insertion into the membrane (115). Oligomerization is a complicated mechanism that involves toxin contact with receptors and subsequent proteolysis of the α-1 helix (116). Activated toxins bind to a wide range of receptors on midgut epithelial cells. The interaction of Cry toxin with its receptor results in toxin oligomerization and pore formation, eventually leading to cell death (117). Sequential binding of Cry1A toxins has been observed in lepidopteran insects. The binding mechanism may begin with alkaline phosphatase (ALP) and aminopeptidase-N (APN) receptors, followed by cadherin binding. Interaction with the cadherin receptor causes α-1 helix to be cleaved, resulting in the formation of oligomeric toxins (116). In case of Cry11Aa, it was reported that Cyt1Aa induce oligomerization of Cry11Aa resulting in membrane pore formation in *Ae. aegypti* (118). Cadherin receptor is important for the oligomerization of Cry11Aa but not for Cry4Ba (119).Cry toxins are very toxic to mosquito larvae. By binding to protein receptors on the gut epithelial cell membrane Cry toxins lead to pore formation and cell lysis (27, 120). Midgut proteins present in the brush border of larvae midgut bind to Cry toxins and facilitate events resulting in larval death (121–123). Many receptors have been reported in mosquitoes, including aminopeptidase (APN), alkaline phosphatase (ALP), cadherin (CAD) and ABC transporters, which are midgut receptors of *Bti* Cry4Ba, Cry11Aa, and Cry11Ba toxin in *Ae. aegypti*, respectively (30, 124–126).

Three conserved signaling pathways, including the Toll-like receptor pathway, immunodeficiency (IMD) pathway, and other Janus kinase-signal transducer and activator of transcription (JKT) pathways, participate in the mosquito defense mechanism (127, 128). The Toll pathway plays main role in the regulation of natural immunity. It is primarily responsible for the identification and protection of viruses and fungi. The IMD pathway can recognize and immunize gram-positive and -negative bacteria and can control antimicrobial peptides, such as Diptera and Drosophila peptides. Expression of AMP (129, 130) and the JKT pathway play important roles in the process of damage repair and tissue regeneration in the body.

## Role of Lectins

Lectins are a class of multivalent proteins that specifically bind glycoproteins and are widely distributed in plants, animals, and microorganisms (35, 37). Lectins play important roles in cell signaling and photosynthesis, and many diverse lectin roles have been studied in the model plant *Arabidopsis thaliana* (131). Recently, plant lectins have been used in agricultural improvement, biomedical research, and glycobiology (132). In animals, they function as weapons to kill pathogens through aggregation and opsonization, and are present in all vertebrates and invertebrates (133, 134). C-type mannose-binding lectin (MBL) plays a key role in the immune system of vertebrates, and its deficit increases the chances of more infectious diseases to attack (41). The MBL in chickens can be activated when they are exposed to chicken diseases (135). Lectins are effective for invertebrate and vertebrate cancerous cells, prompting biochemists to use them in histochemical and cytochemical research (136, 137) as well as in human medicine (138)

### Role of Lectins in Insects

Insects are a very abundant and miscellaneous phylum in the kingdom Animalia. They rely entirely on their innate immune system to prevent themselves from external environmental pathogens (42, 139, 140). When a harmful germ invades an insect body, it is recognized by a group of proteins recognized as pattern recognition receptors (PRRs). These PRRs can detect pathogens *via* the pathogen-associated molecular patterns (PAMPs) present on the pathogen surface (46). Invertebrates have seven groups of PRRs, namely, galactose-binding lectins (galectins), multi-domain scavenger receptors (SCRs), peptidoglycan recognition proteins (PGRPs), fibrinogen-related proteins (FREPs), gram-negative binding proteins (GNBPs), thioester-containing proteins (TEPs), and CTLs. More recently, Toll-like receptors and the mammalian Toll receptor family have been found to be more conserved and to function in innate immunity. *Bombyx mori* Toll9 acts as a PRR for lipopolysaccharide binding and Toll9 is more similar to the mammalian TLR4–MD-2–LPS pathway (141).

CTLs are a large family of proteins that are recognized by *CTLDs* and further classified into 17 different subgroups on the basis of structural domain and phylogeny (44). They bind carbohydrates in the presence of Ca^2+^ ions *via* their *CTLD*, containing the highly conserved motifs *EPN* (Glu-Pro-Asn) and *QPD* (Gln-Pro-Asp), which are specific to mannose-and galactose-type carbohydrates (43, 142). CTLs exhibit a wide range of functional responses in symbiosis, host colonization by infectious pathogens, and host immune responses (36, 41). Invertebrate CTLs have been shown to mediate immune responses and development (143, 144). Innate immunity is based on the secretion of different lectins that possess different functions, including nodule formation, *Escherichia coli* clearance, hemagglutination, encapsulation, melanization, the prophenoloxidase cascade, and phagocytosis (145, 146).

The novel CTLs *TcCTL5* and *TcCTL6* in the Coleopteran beetle (*Tribolium castaneum*) functioned against bacterial infection, whereas their silencing showed a significant decrease in four antimicrobial peptides (147, 148). A CTL in *Plutella xylostella*, *PxIML*, play a key role in the recognition of pathogen and the subsequent humoral and cellular immunity of the species (39). Similarly, the Mud Crab (*Scylla paramamosain*) CTL *SpCTL6* plays an immune-protective role, and its expression level is significantly increased during the larval stages and after molting (149). A genome-wide comparative analysis of CTLs in seven insect species (*Spodoptera litura*, *Helicoverpa armigera, Manduca sexta, B. mori, Drosophila melanogaster, Tribolium castaneum*, and *Ae. mellifera*), showed interesting results. They observed that CTL-S1–S8 and CTL-X1–X4 ortholog groups were well conserved in seven species, whereas the CTL-X5 double CRD domain group, the three-CRD CTL-S11 group, the C-terminal long CTL-S9 group, and the CTL-Lepidopteran-specific S10 group were found to be not conserved (150). Furthermore, the CTL *BrCTL10* induces multiple immune responses in silkworms (*B. mori*) (40). In addition, *BmLBP* in *B. mori* facilitates the clearance of *E. coli* (151). Most importantly, these insect CTLs can recognize dead cells as well as cancerous cells in invertebrates (152, 153). A total of 35 CTL genes were identified in the Oriental Armyworm *(Mythimna separate)* with a single and double CRD domain that roles in innate immune responses (154). *M. sexta* immulectins enable melanization and cellular encapsulation (155, 156). Furthermore, *HaCTL3*, a CTL gene in the cotton bollworm *(H. armigera)* plays a key role in development and larval growth (157).

### Role of Lectins in Mosquitoes

The mosquito’s gut is responsible for pathogen entry and replication. The gut contains microbiome that interact with midgut cells and are essential for vector physiology (158, 159). Previous studies reported that the gut microbiome plays a vital role in vector competence (158, 160–162). CTLs in gut ecology play a vital role in immune activation and may serve as intervention targets for the control of vector-borne diseases in nature (36, 163). *Ae. aegypti mosGCTL-3* regulates germline development and affects fertility, whereas knockout of *mosGCTL-3* revealed a decrease in the number of gut microbiota, and *GCTL-3* mutants showed a decrease in the dengue virus-2 infection rate (164). Modification of the mosquito’s immune system through expression of the human CTL *CLEC18A* gene can drastically reduce dengue virus infection. Transgenic mosquitoes showed significant differences in the midgut microbiota (165). Mosquito galectin, *mosGCTL-1*, interacts with the West Nile virus (WNV) and promotes mosquito infection (166) while *mosGCTL-7* interacts with the Japanese encephalitis virus (JEV) in *Ae. aegypti* and facilitates virus entry (167).

**Figure 2:**
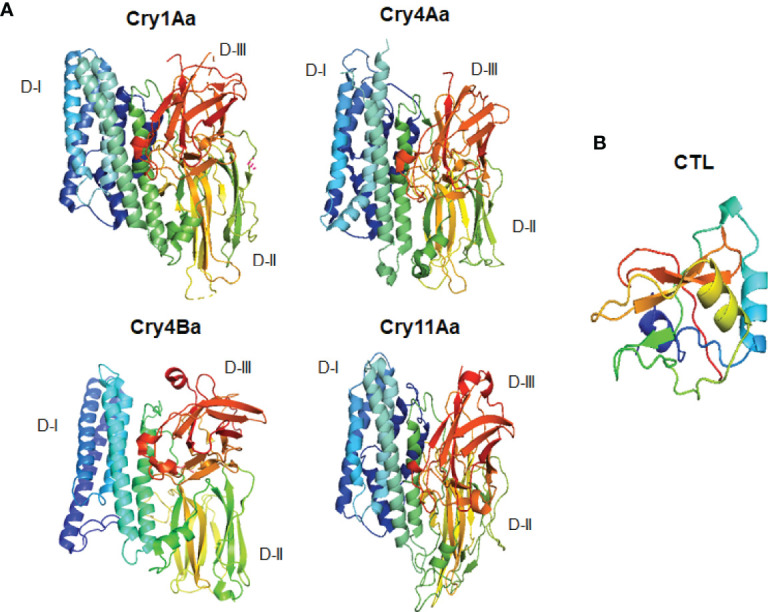
Three dimensional structural comparisons between different Cry toxins and CTL domain. **(A)** Cry1Aa (PDB: D6J4), Cry11Aa (PDB: 1DLC), Cry4Aa (PDB: 2C9K), Cry4Ba (PDB: 1W99); **(B)** CTL domain (PDB: 5E4L).

The mosquito genomes of *Ae. aegypti* and *Anopheles gambiae*, and those of *D. melanogaster* and *M. sexta*, contain 39, 25, and 34 CTL genes, respectively (45, 127, 168, 169), whereas 183 CTL genes have been reported in *Caenorhabditis elegans* (168). Mosquito, shrimp, and Drosophila CTLs help these species defend themselves against bacterial infections. It has also been reported that silencing of CLTs causes rapid bacterial growth in cases of infection, which ultimately results in a short lifespan (170, 171). Furthermore, it has been described that mosquito CTLs play functions in the maintenance of homeostasis of the gut microbiome (36). CLTs play significant role in the activation of the melanization cascade in *Ae. aegypti* (172). Moreover, the CRDs of lectins and the *Bti* Cry toxin domains II and III adopt similar structures (48–50, 173). The tertiary structures of different *Bti* Cry toxins have determined through X-ray crystallography (27) (**Figure 2**). All of these structures are very similar to the three-domain organization, suggesting that all proteins in the Cry three-domain family share a similar mode of action. The N-terminal domain (domain I) consists of seven α helices, the central -α5 helix is ​​hydrophobic and surrounded by six additional amphipathic helices; the helical domain is necessary for membrane insertion and pore formation. Domain II is made up of three anti-parallel β-sheets with exposed loop sections, while domain III is made up of a β-sandwich (174, 175). In domains II and III, exposed regions are required for receptor binding (27). Domain II shares structural resemblances with various carbohydrate-binding proteins, including lectin jacalin, lectin Mpa and vitelline (59, 176–179); Domain III is structurally identical to other carbohydrate-binding proteins like the cellulose binding domain of 1,4-β-glucanase C, β-glucoronidase, β-galactosidase, galactose oxidase, sialidase, and xylanase U (180). Because of these similarities, carbohydrate moieties may play a substantial part in the mechanism of three-domain Cry toxins.

Due to various structural similarities, it is very important to further understand the function and molecular mechanism of mosquito lectin in Cry toxicity, protein-protein interactions among lectin, Cry toxin, and other important receptors (51, 52, 54, 125, 173).

### Role of Lectins in the *Ae. aegypti* Response Against Cry Toxin

Cry toxin tolerance, especially Cry1A, has been extensively studied in Lepidoptera such as *B. mori*. Cry1A toxicity is altered in the presence of the midgut protein P252 and has antimicrobial activity against *Bt, E. coli*, and *Serratia marcescens* (181). These midgut membrane proteins also show low toxicity of Cry1Ac in *H. armigera* (182). In other Lepidopteran larvae, like *Lymantria monacha*, *Thaumetopoea pityocampa*, *Heliothis virescens*, *M*. *sexta*, and *Spodoptera exigua*, decrease Cry toxicity in late instars is associated with a decreased number of available binding sites (25, 183–186). Weaker interaction of Cry1A toxins was identified among the apical brush border of the midgut epithelium of *Orgyia pseudotsugata* and Cry1A toxins due to the presence of toxin-binding glycoproteins in the larval midgut (55). In *M. sexta*, Cry1Ac binding to the APN receptor is inhibited by the presence of N-acetylgalactosamine (GalNAc) on the receptor and decreases Cry toxicity. The Cry-domain III folds are involved in receptor recognition of carbohydrates, and GalNAc binds to Cry1Ac domain III positions and plays a competitive role like the lectin domain (56, 187).

**Figure 3:**
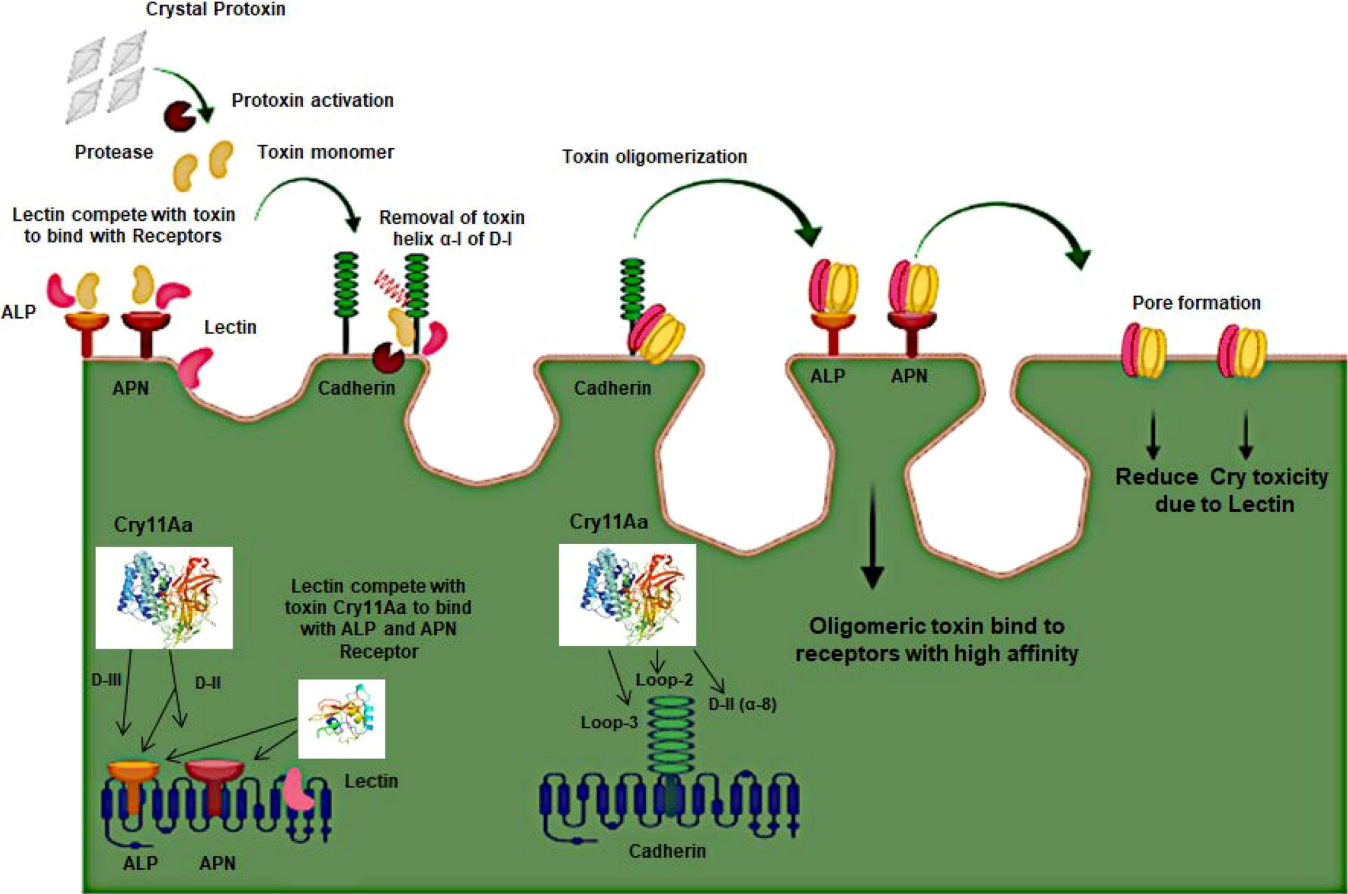
Schematic presentation of the 3D-Cry toxin mechanism with Receptors and Lectin in Mosquito.

**Figure 4:**
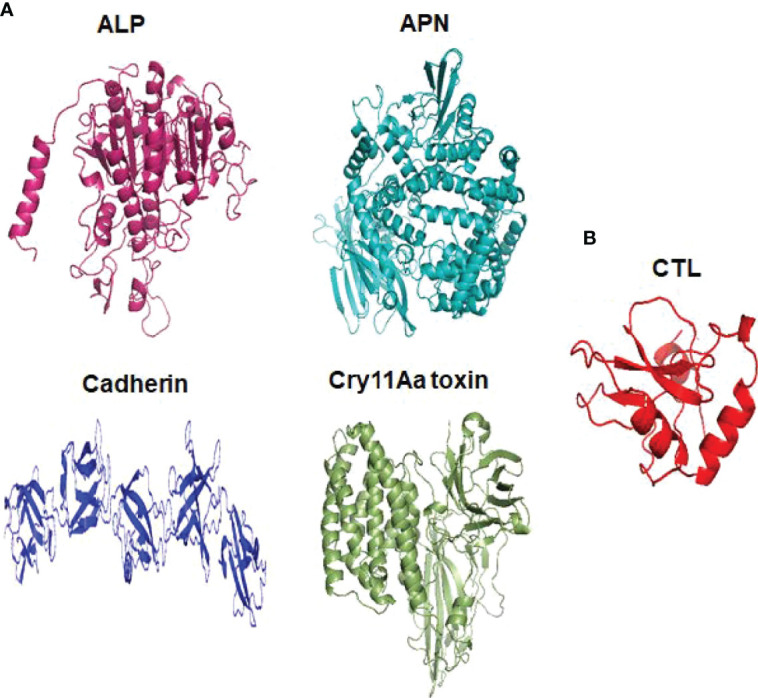
Three dimensional structural presentation of putative receptors, toxin and CTL domain in *Ae. aegypti*. **(A)** ALP (PDB: IK7H), APN (PDB: 4WZ9), Cadherin (PDB: 4UX8), Cry11Aa toxin (PDB: 1DLC) and **(B)** CTL domain (PDB: 5E4L).

**Figure 5:**
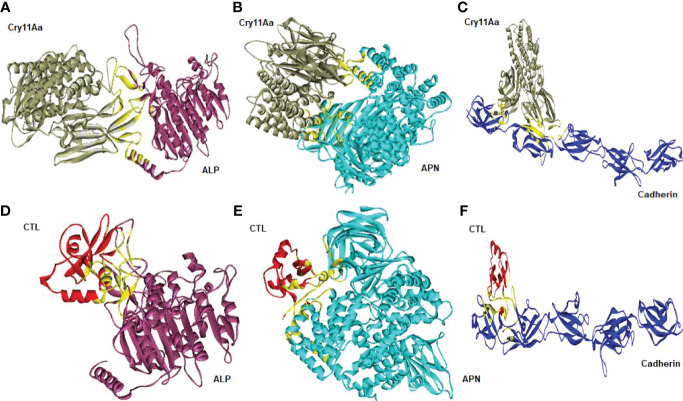
Molecular docking representation of receptors with Cry11Aa and CTL proteins. Cry11Aa binding with **(A)** ALP, **(B)** APN, and **(C)** Cadherin receptors. CTL binding with **(D)** ALP, **(E)** APN, and **(F)** Cadherin receptors. Yellow color showed the binding sites of two proteins.

**Figure 6:**
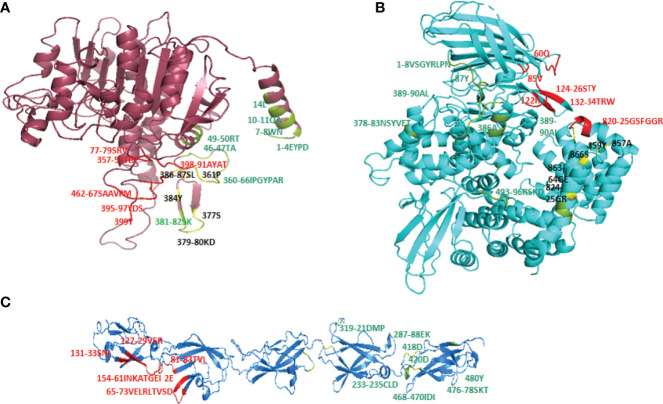
Overlapping binding sites in receptors interface. When both CTL and Cry11Aa proteins docked together in ALP, APN and Cadherin receptors overlapping binding sites (colored in yellow) were found in **(A)** ALP and **(B)** APN but none of residue in Cadherin receptor **(C)** found to be overlapped. Red colored residues are CTL binding sites while Cry11Aa binding sites are colored in green smudge.

Cry toxins bind to putative receptors, including ALP, APN, and CAD in the midgut epithelium of *Ae. aegypti*. ALP contains at least two Cry11Aa binding sites, such as residues R59-G102 interacting with loop α-8 from Cry11Aa domain II, and residues N257-I296 interacting with domain III of Cry11Aa (26, 124). The full-length AaeAPN2 region, including amino acids 569–641, has the highest binding activity to the Cry11Aa toxin and efficiently competes with the toxin binding to *Aedes* BBMV (54). The cadherin fragment, which contains CR7–11 (cadherin repeats 7–11) binds to Cry11Aa, primarily through loop α8 of domain II toxin, while Loop-3 of Cry11Aa binds to CR11 (cadherin repeats) of *Ae. aegypti* (51). Midgut proteins play an important role in this toxicity mechanism and alter the binding activity with receptors and Cry toxins. Previously, we identified highly expressed C - and G-type lectins in the *Ae. aegypti* midgut after treatment with the *Bt* LLP29 toxin (176). These midgut CTLs and galectins have been reported to inhibit Cry11Aa toxicity in *Ae. aegypti* by competing with Cry11Aa for binding to ALP and APN receptors (176–178) (**Figure 3**), but no evidence of binding competition was found in the case of CAD (177). Further silencing of these midgut proteins results in enhanced toxicity of Cry toxins (177). Moreover, the three-dimensional protein structures of the putative receptors ALP, APN, CAD, Cry11Aa toxin, and CTL were modeled in previously reported study (177–179) (**Figures 4A, B**). Molecular docking of ALP, APN, and CAD with both Cry11Aa and CTL showed that all receptors were docked to the CTL and Cry11Aa (178), and the residues (yellow colored) were the binding sites of the two proteins (**Figures 5A–F**). Even when these two proteins docked together with ALP and APN receptors, overlapping binding sites were found where residues in Cry11Aa and CTL were competing to bind with receptors (overlapping sites colored in yellow) (177, 178). Residues in red are the CTL binding sites, while the green smudge regions are the Cry11Aa binding sites in the ALP and APN receptors (**Figures 6A, B**) (178). However, no overlapping sites were found when CTLs and Cry11Aa were docked with the CAD receptor (179) (**Figure 6C**). It was suggested that these important proteins could sequester the toxin and interfere with the insecticidal process. Furthermore, the fact that these proteins are immune-related may suggest that Cry toxins may alter may alter insect’s immune responses. Such compounds or chemicals should be introduced to counteract the effect of CTLs in the midgut and improve the toxicity mechanism. These interesting ideas warrant future studies.

## Concluding Remarks and Future Perspectives

To date, many researchers have focused on the identification of different lectins and their further characterization in different organisms. Lectins have also been well studied in higher organisms, such as plants and animals, but limited literature is available on insects. Lectins play a crucial role in the innate immunity of insects. Both invertebrate and vertebrate CTLs contain specific CRDs. Nevertheless, research into the mechanisms and actions of insect CTLs in innate immunity will contribute to the protection of beneficial insects as well as the biological control of harmful vectors. Therefore, it is important to study the role of lectins in mosquitoes, especially in *Ae. aegypti.* Thus, if the major interaction among toxins and their receptors is reduced or eliminated, the toxicity of *Bt* will be greatly altered. Midgut protein engineering may also a considerable way to improve Cry toxicity. The expression of *Ae. aegypti* galection-14 was knocked down which resulted in increasing Cry toxicity (177). Still, the molecular studies in this domain are limited and need more experimental evidence in mosquitoes and other species. On another side, many reports published showed improving Cry toxins activity against mosquitoes and insects by using recombinant Cry toxins (188). Several reported studies have shown that midgut proteins may influence Cry toxin activity and have been studied in many other insect species, including *P. xylostella* (193), *Trichoplusia ni* (194), *Leptinotarsa decemlineata* (195), *Cnaphalocrocis medinalis* (196), *Achaea janata* (197), and the insect family Noctuidae (198). Therefore, the detection and identification of important midgut proteins that may interfere with this critical step may open a new avenue of research to fully understand the *Bt* mechanism and give a theoretical foundation for the development of new bioinsecticides for mosquito control.

## Author Contributions

The review of literature, and manuscript writing were accomplished by IA and KB. WT, AI, XG and LZ revised the manuscript. XG and LZ provided technical support and vigorous guidance, and founded the research project. The authors read and approved the final manuscript.

## Funding

This work was funded by the National Program of China (Grant Numbers 2017YFE0121700 and 2017YFE0122000); the United Fujian Provincial Health and Education Project for Tackling Key Research (Grant No. 2019-WJ-29); Natural Science Foundation of Fujian Province (Grant No. 2020J01550 and 2020I0031); the Special Fund for Scientific and Technological Innovation of Fujian Agriculture and Forestry University (KFA20124A).

## Conflict of Interest

The authors declare that the research was conducted in the absence of any commercial or financial relationships that could be construed as a potential conflict of interest.

## Publisher’s Note

All claims expressed in this article are solely those of the authors and do not necessarily represent those of their affiliated organizations, or those of the publisher, the editors and the reviewers. Any product that may be evaluated in this article, or claim that may be made by its manufacturer, is not guaranteed or endorsed by the publisher.
